# Rosmarinic Acid Potentiates Cytotoxicity of Cisplatin against Colorectal Cancer Cells by Enhancing Apoptotic and Ferroptosis

**DOI:** 10.3390/life14081017

**Published:** 2024-08-15

**Authors:** Jhen-Yu Huang, Ta-Wen Hsu, Yu-Ru Chen, Shao-Hsuan Kao

**Affiliations:** 1 Institute of Medicine, College of Medicine, Chung Shan Medical University, Taichung 402306, Taiwan; mrhuang55@gmail.com (J.-Y.H.); qweqwe789789741741102@gmail.com (Y.-R.C.); 2 Division of Colorectal Surgery, Buddhist Tzu Chi Medical Foundation, Dalin Tzu Chi Hospital, Chiayi 622401, Taiwan; b120018@tzuchi.com.tw; 3 School of Medicine, Tzu Chi University, Hualien 970374, Taiwan; 4 Department of Medical Laboratory and Biotechnology, Chung Shan Medical University, Taichung 402306, Taiwan; 5 Department of Medical Research, Chung Shan Medical University Hospital, Taichung 402306, Taiwan

**Keywords:** rosmarinic acid, cisplatin, colorectal cancer cell, apoptosis, ferroptosis

## Abstract

Rosmarinic acid (RA) has demonstrated anticancer effects on several types of malignancies. However, whether RA promotes the anticancer effect of cisplatin on colorectal cancer cells remains sketchy. This study aimed to explore whether RA potentiates the cytotoxicity of cisplatin against colon cancer cells and the underlying mechanism. Cell viability, cell cycle progression, and apoptosis was evaluated using sulforhodamine B (SRB) assay, flow cytometric analysis, and propidium iodide/Annexin V staining, respectively. Western blotting was utilized to analyze signaling pathways. Our findings showed that RA significantly enhanced the inhibitory effect on cell viability and the induction of apoptosis on the colon cancer cell lines DLD-1 and LoVo. Signaling cascade analysis revealed that the combination of RA and cisplatin jointly induced Bax and caspase activation while downregulating Bcl-2, glutathione peroxidase 4 (GPX4), and SLC7A11 in DLD-1 cells. Moreover, caspase inhibitor and ferroptosis inhibitor significantly reversed the inhibition of cell viability in response to RA combined with cisplatin. Collectively, these findings demonstrate that RA enhances the cytotoxicity of cisplatin against colon cancer cells, attributing to the promotion of apoptosis and ferroptosis.

## 1. Introduction

Colorectal cancer (CRC) remains a leading cause of cancer-related mortality worldwide, with over 900,000 deaths reported annually [1]. CRC often arises from precancerous growths called adenomatous polyps, which can undergo malignant transformation into adenocarcinoma due to the accumulation of genetic mutations that drive excessive cell proliferation and impair programmed cell death such as apoptosis in the epithelial cells lining the colorectal tract [2]. While early detection of colorectal cancer can significantly improve clinical outcomes for patients, the 5-year survival rate remains alarmingly low for those diagnosed with advanced stage III or stage IV disease [3]. The unfavorable prognosis and low survival rates observed in colorectal cancer patients can be largely attributed to incomplete elimination of cancer cells during treatment, the development of resistance to chemotherapeutic agents, and the metastatic spread of cancer cells to distant organs and tissues [4].

Cisplatin is a platinum-based chemotherapeutic agent widely utilized in cancer treatment regimens [5]. It is one of the standard chemotherapeutic drugs, along with 5-fluorouracil, for the management of colon cancer. Cisplatin exerts its anticancer effects through multiple mechanisms, including inhibition of DNA synthesis, generation of DNA lesions, and induction of mitochondrial apoptosis via the formation of DNA adducts with its platinum atoms [6]. However, a significant clinical challenge with cisplatin is the development of resistance mechanisms in cancer cells, rendering them less susceptible to the antiproliferative and cytotoxic effects of the drug. Consequently, a substantial proportion of patients treated with cisplatin experience therapeutic failure and tumor recurrence. Additionally, the use of cisplatin is further limited by its notable adverse effects and toxicities [7]. To address these limitations, increasing research efforts have focused on identifying adjuvant drugs that can be combined with cisplatin to potentiate its antineoplastic effects while concurrently minimizing its toxicity and side effects.

Combination therapy with natural compounds that can potentiate the anticancer effects of cisplatin while minimizing toxicity represents a promising strategy to improve treatment outcomes [8,9]. Rosmarinic acid (RA), a polyphenolic compound abundant in herbs such as rosemary, mint, and basil, has garnered significant attention for its diverse pharmacological properties, including antioxidant, anti-inflammatory, and anticancer activities [10]. Accumulating evidence suggests that RA exhibits anticancer effects against various malignancies, such as breast [11], lung [12], and pancreatic cancers [13], through modulation of multiple signaling pathways involved in cell proliferation, apoptosis, and metastasis. However, the potential synergistic effects of RA in combination with cisplatin on colorectal cancer cells and the underlying mechanisms remain largely unexplored.

In this study, we investigated whether RA could enhance the cytotoxicity of cisplatin against CRC cell lines. The effects of the combination treatment on cell viability, cell cycle progression, and programmed cell death pathways were evaluated. These findings provide novel insights into the anticancer effects of RA and cisplatin on CRC cells, highlighting the potential of this combination as a therapeutic strategy.

## 2. Materials and Methods

### 2.1. Reagents and Antibodies

Cisplatin, dimethyl sulfoxide (DMSO), sulforhodamine B (SRB), Tris base, and nonspecified reagents were obtained from Sigma-Aldrich (St. Louis, MO, USA). Rosmarinic acid (RA) was purchased from Cayman Chemical (Ann Arbor, MI, USA). Dulbecco’s-modified Eagle’s medium (DMEM) and fetal bovine serum (FBS) were obtained from Gibco (Grand Island, NY, USA). Antibodies against human Bcl-2 (sc-7382), β-actin (sc-8432), and peroxidase-conjugated secondary antibodies against mouse or rabbit IgG (sc-516102, sc-2357) were acquired from Santa Cruz Biotechnology (Santa Cruz, CA, USA). Antibodies against human cleaved caspase-3 (#9664), cleaved caspase-9 (#7237), Bax (#2774), GPX4 (#52455), SLC7A11 (#12691), and NCOA4 (#66849) were purchased from Cell Signaling Technologies (Beverly, MA, USA).

### 2.2. Cell Culture and Treatments

DLD-1 and LoVo, human colon cancer cell lines, were procured from BCRC (Hsinchu, Taiwan) and maintained in RPMI-1640 medium complemented with 10% fetal bovine serum (FBS, Biological Industries, Cromwell, CT, USA) at 37 °C with 5% CO_2_. Cells that reached 80% confluency were collected and used for treatments with specified concentrations of RA, cisplatin, or their combinations for the indicated times. Post-treatment, the cells were washed with PBS and then harvested for further analyses. PBS was utilized as the solvent control during treatment.

### 2.3. Cytotoxicity Assay

Cytotoxicity was evaluated using the SRB assay as previously described [14]. Briefly, cells were seeded in 24-well plates and treated with various concentrations of RA, cisplatin, or their combination for 24 h. After treatment, cells were fixed with trichloroacetic acid and stained with SRB solution. After being washed with 1% acetic acid, the plates were dried in air. The protein-bound dye was solubilized with 10 mM Tris buffer (pH 10.5), and the absorbance at 510 nm was measured using a microplate reader.

### 2.4. Cell Cycle Analysis and Cell Apoptosis Assay

Cell cycle distribution was evaluated using propidium iodide (PI) staining and flow cytometric analysis. Following exposure to RA, cisplatin, or a combination of both for 24 h, cells were collected and fixed with ice-cold 70% ethanol. Subsequently, they were centrifuged at 600× *g* for 5 min, treated with 100 mg/mL RNase in PBS for 30 min at 37 °C, and stained with 50 mg/mL PI in PBS. Cell cycle distribution was determined using a FACS Calibur system (version 2.0, BD Biosciences, Franklin Lakes, NJ, USA) equipped with CellQuest software (version 5.1).

Cell apoptosis was assessed by using BD Pharmingen™ PE Annexin V Apoptosis Detection Kit (BD Biosciences, San Jose, CA, USA) according to manufacturer’s instruction. Briefly, cells (5 × 10^5^) were exposed to adenine, cisplatin, or the adenine–cisplatin combination for 24 h, resuspended in 500 μL binding buffer, and then incubated with Annexin V–fluorescein isothiocyanate (FITC; 5 µL) and PI (5 µL). After incubation in the dark for 30 min, cells were analyzed using the FACS Calibur system. Cells stained only with Annexin V–FITC indicated early apoptosis, while cells showing both Annexin V–FITC and PI staining signals indicated late apoptosis. Cell apoptosis was presented as percentage of cells with early or late apoptosis.

### 2.5. Protein Extraction and Western Blot Analysis

Cell lysis for protein extraction was conducted using RIPA buffer supplemented with protease and phosphatase inhibitor cocktail (Sigma-Aldrich) at 4 °C for 30 min. Following centrifugation at 20,000× *g* at 4 °C for 15 min to eliminate insoluble debris, the resulting supernatant was served as crude protein extract and subjected to protein quantitation assay by using the Bradford method according to the manufacturer’s instructions (Bio-Rad Laboratories, Hercules, CA, USA).

For Western blotting, 20 µg crude protein for each sample was subjected to SDS-polyacrylamide gel electrophoresis, followed by transfer onto a polyvinylidene difluoride membrane (Immobilon, Merck, Darmstadt, Germany). The membrane was then blocked with 5% skimmed milk in PBS for 1 h and subsequently probed with primary antibodies for 2 h. After being washed with PBS containing 0.5% Tween-20 (PBST), the membrane was incubated with secondary antibodies for 2 h, and then the bound secondary antibodies were determined using an ECL chemiluminescence reagent (LumiFlash Ultima Chemiluminescent Substrate; Visual Protein, Taipei, Taiwan), and the resulting chemiluminescence signals were captured and semiquantified using an image analysis system (Fujifilm, Tokyo, Japan). PBS treatment signals served as the control.

### 2.6. Statistical Analysis

All experiments were performed in triplicate, and data are expressed as mean ± standard deviation (SD). Statistical analyses were conducted using SPSS for Windows version 25 (IBM Corp., Armonk, NY, USA). Differences between groups were analyzed using Student’s *t*-test or one-way ANOVA followed by Tukey’s post hoc test. A *p*-value < 0.05 was considered statistically significant.

## 3. Results

### 3.1. RA Enhanced the Inhibitory Effect of Cisplatin on Cell Viability of DLD-1 and LoVo Cells

**Figure 1:**
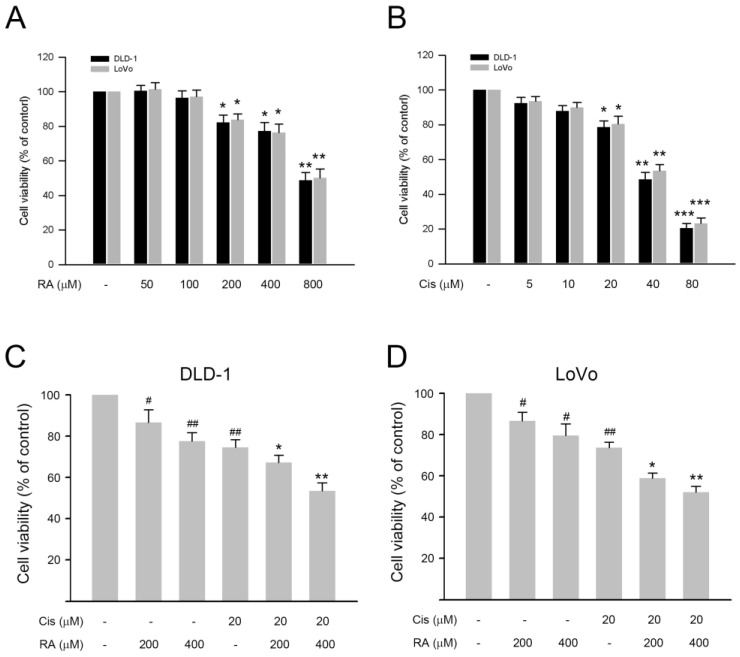
Inhibitory effects of RA and the combination of RA with cisplatin on cell viability of colon cancer cells. Cells were treated with RA, cisplatin (Cis), or their combinations for 24 h, followed by subjecting the treated cells to SRB assay to evaluate cell viability. Cell viability was presented as percentage of control. (**A**) Effect of RA on cell viability of DLD-1 ad LoVo cells, (**B**) Effect of cisplatin on cell viability of DLD-1 ad LoVo cells, and (**C**,**D**) Effect of RA combined with cisplatin on cell viability of DLD-1 and LoVo cells. For (**A**,**B**) *, **, and ***, *p* < 0.05, *p* < 0.01, and *p* < 0.005 vs. control. For (**C**,**D**), # and ##, *p* < 0.05 and *p* < 0.01 vs. control, respectively. * and **, *p* < 0.05 and *p* < 0.01 vs. 20 μM cisplatin treatment alone, respectively.

First, the inhibitory effects of RA and cisplatin on DLD-1 and LoVo cells were explored. Our observations showed that both RA (50–800 µM) and cisplatin (5–80 µM) reduced the cell viability of DLD-1 and LoVo cells in a dose-dependent fashion (Figure 1A,B). Notably, when administered at the highest tested dosage, 800 µM RA and 80 µM cisplatin dramatically decreased the cell viability to approximate 50% and 20% of control, respectively (Figure 1A,B, *p* < 0.01). Then, the inhibitory effects of the combination of RA and cisplatin on both CRC cells were assessed. As shown in Figure 1C,D, the combination of RA (400 µM) and cisplatin (20 µM) significantly reduced the cell viability of DLD-1 and LoVo cells to around 53% and 51% of the control, respectively (*p* < 0.01 vs. 20 µM cisplatin alone). Compared to that, 400 µM RA and 20 µM cisplatin reduced the cell viability to around 72% and 76%, respectively; the combination of RA and cisplatin exhibited stronger inhibitory effects on the CRC cells. Together these findings indicate that RA potentiates the cisplatin-induced inhibition of cell viability in CRC cells.

### 3.2. RA Combined with Cisplatin Promoted Cisplatin-Induced Sub-G1 and S Phase Accumulation in DLD-1 and LoVo Cells

**Figure 2:**
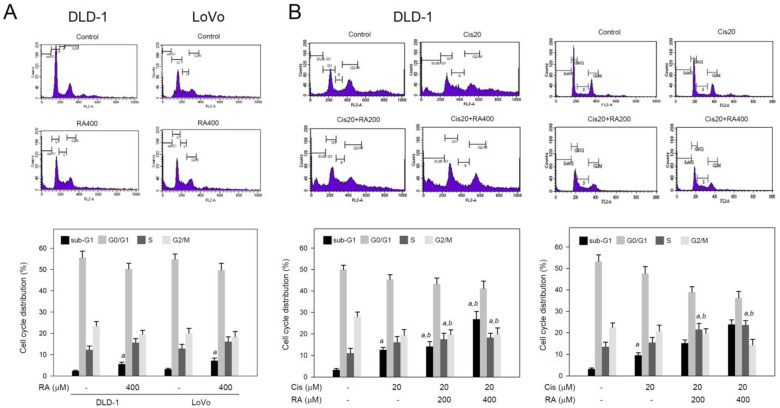
Effects of RA, cisplatin, and the combination of cisplatin with RA on cell cycle distribution in colon cancer cells. Cells were treated with (**A**) 400 μM RA alone or (**B**) cisplatin (Cis), RA, or their combinations for 24 h, and then the treated cells were fixed and stained with PI. Flow cytometric analysis was used to determine the ratios of cells in individual cell cycle phase. *a* and *b*, *p* < 0.05 vs. control (-) and 20 μM cisplatin treatment alone, respectively.

Next, the influence of RA combined with cisplatin in cell cycle progression was explored. As shown in Figure 2A, RA (400 μM) alone elevated the sub-G1 phase ratio from 2% (control) to 5% and 8% for DLD-1 and LoVo cells, respectively (*p* < 0.05). In contrast to the elevation of sub-G1, RA alone did not significantly affect the S phase ratio in both cells. For the combination, 20 µM cisplatin resulted in sub-G1 and S phase accumulation in DLD-1 and LoVo cells (Figure 2B). Of important, the combination of RA and cisplatin significantly increased the sub-G1 accumulation in DLD-1 and LoVo cells approximately from 3% and 2% (cisplatin alone) to 28% and 22% (cisplatin + RA), respectively. Similarly, the combination of RA and cisplatin significantly also increased the S phase accumulation in DLD-1 and LoVo cells from 15% and 16% (cisplatin alone) to around 18% and 25% (cisplatin + RA), respectively (Figure 2B). These findings indicate that RA increases the sub-G1 and S phase accumulation in response to cisplatin stimuli. It suggests that RA may enhance the cisplatin-induced apoptosis evidenced by sub-G1 accumulation and raise the cisplatin-triggered cell cycle arrest at S phase evidenced by S phase accumulation.

### 3.3. RA Enhanced Cisplatin-Induced Apoptosis in CRC Cells

**Figure 3:**
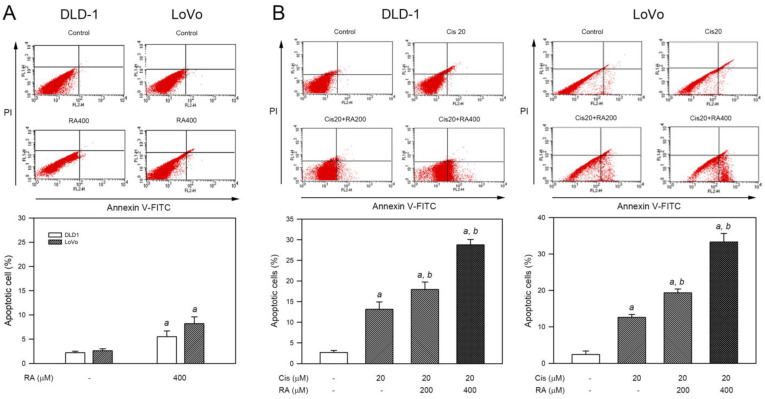
Induction of apoptosis by RA, cisplatin, and the combination of cisplatin with RA in colon cancer cells. Cells were treated with (**A**) 400 μM RA alone or (**B**) cisplatin (Cis), RA, or their combinations for 24 h. The cells that received treatment were fixed, stained with PI and Annexin V-FITC (ANXV), and then analyzed using flow cytometry to calculate the proportions of cells showing PI-positive, ANXV-positive, or both characteristics. *a* and *b*, *p* < 0.05 vs. control and 20 μM cisplatin treatment alone, respectively.

Since sub-G1 accumulation implicates apoptosis, the ability of RA, cisplatin, and RA combined with cisplatin to induce apoptosis was evaluated using Annexin V-FITC/PI staining and flow cytometry analysis. As shown in Figure 3A, RA (400 μM) alone increased the average percentage of apoptotic cells from 2.3% to 5.6% and from 2.9% to 7.5% for DLD-1 and LoVo cells, respectively (*p* < 0.05). For the combination, treatment with 20 μM cisplatin alone for 24 h moderately increased the average percentage of apoptotic cells from 2.7% to 13.1% (DLD-1) and from 2.1% to 14.2% (LoVo), respectively (*p* < 0.05 vs. control group in both cell lines). Notably, the combination of RA (200 or 400 μM) and cisplatin (20 μM) significantly enhanced apoptosis induction, leading to the elevation of the average percentage of apoptotic cells up to 28.8% (DLD-1) and 33.6% (LoVo), respectively (*p* < 0.05 vs. cisplatin alone in both cell lines). Collectively, these findings indicate that RA promotes the apoptosis of CRC cells provoked by cisplatin.

### 3.4. RA Enhanced the Apoptotic Signaling Induced by Cisplatin in DLD-1 Cells

**Figure 4:**
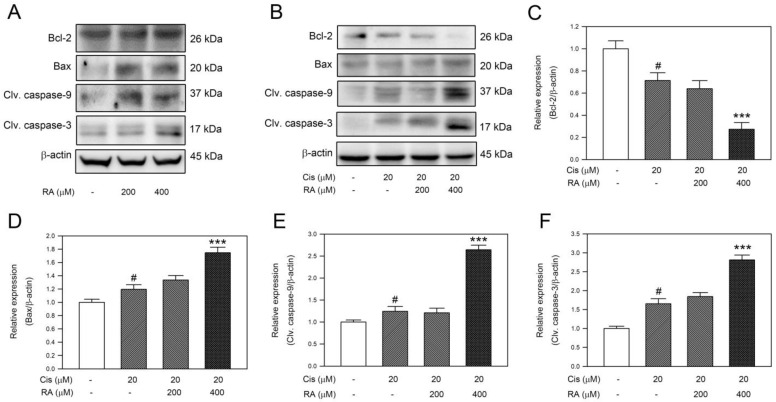
RA and its combination with cisplatin reduced antiapoptotic Bcl-2 and enhanced proapoptotic signals in DLD-1 cells. Cells were treated with RA, cisplatin, and the combination of cisplatin with RA for 24 h, and then the cells were subjected to (**A**,**B**) assess the levels of antiapoptotic Bcl-2 and proapoptotic Bax, cleaved (clv.) caspase-9, and clv. caspase-3 by Western blotting. Relative quantitation was conducted by using densitometric analysis, and the results are shown in (**C**–**F**). Level of β-actin was used as internal control. #, *p* < 0.05 vs. control. ***, *p* < 0.005 vs. 20 μM cisplatin treatment alone.

Based on the findings that cisplatin and RA significantly trigger cell apoptosis in CRC cells, the apoptotic signaling components were further assessed in DLD-1 cells. As shown in Figure 4A, antiapoptotic protein Bcl-2 was not influenced by RA alone. In contrast to Bcl-2, RA upregulated proapoptotic Bax and induced activation of caspase-9 and caspase-3. For the combination, Bcl-2 was downregulated by cisplatin treatment (20 μM, *p* < 0.05 vs. control), and the Bcl-2 downregulation was further promoted by the combination of RA (400 μM) with cisplatin (20 μM) (Figure 4B,C, *p* < 0.005 vs. cisplatin alone). On the contrary, Bax was significantly upregulated by the combination of RA (400 μM) with cisplatin (20 μM) (Figure 4B,D, *p* < 0.005 vs. cisplatin alone). Next, the influence of RA combined with cisplatin in caspase activation was explored, and the results revealed that the levels of cleaved caspase-9 and cleaved caspase-3 were significantly increased in response to 20 μM cisplatin alone and the combination of RA (400 μM) with cisplatin (20 μM) in DLD-1 cells (Figure 4B,E,F). Collectively, these observations indicate that RA enhances the apoptotic signaling induced by cisplatin in DLD-1 cells.

### 3.5. RA Promoted the Ferroptosis Signaling Induced by Cisplatin in DLD-1 Cells

**Figure 5:**
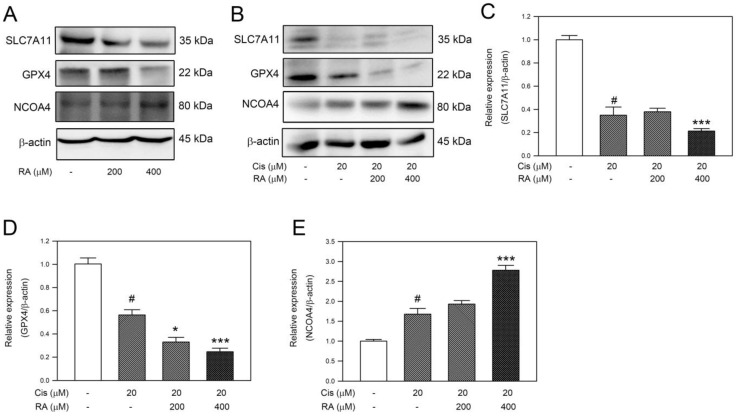
RA and its combination with cisplatin promoted ferroptosis signals in DLD-1 cells. Cells were treated with RA, cisplatin, or the combination of cisplatin with RA for 24 h, and then the cells were subjected to (**A**,**B**) assess the levels of ferroptosis-related components by Western blotting. Relative quantitation was conducted by using densitometric analysis, and the results are shown in (**C**–**E**). Level of β-actin was used as internal control. #, *p* < 0.05 vs. control. * and ***, *p* < 0.05 and *p* < 0.005 vs. 20 μM cisplatin treatment alone, respectively.

Recently, there has been considerable interest in nonapoptotic cell death in tumor therapy due to the fact that cancer often exhibits resistance to apoptosis. Therefore, whether RA and its combination with 20 μM cisplatin triggered ferroptosis-associated signaling was studied in DLD-1 cells. Our observations revealed that RA alone decreased SLC7A11 and GPX4 levels but increased NCOA4 level. Similarly, cisplatin reduced the cellular levels of SLC7A11 and GPX4 while simultaneously increasing the expression of NCOA4 (Figure 5A,B, *p* < 0.05 vs. control). Notably, the combination of 20 μM cisplatin with 400 μM RA reduced the cellular levels of SLC7A11 and GPX4 lower than 20 μM cisplatin alone while elevating the level of NCOA4 higher than 20 μM cisplatin alone (Figure 5B–E, *p* < 0.005 vs. cisplatin alone). Taken together, these findings indicate that RA strengthens the ferroptosis-associated signaling in response to cisplatin in CRC cells.

### 3.6. RA Enhanced Cisplatin-Induced Cytotoxicity through Promoting the Apoptosis and Ferroptosis in CRC Cells

**Figure 6:**
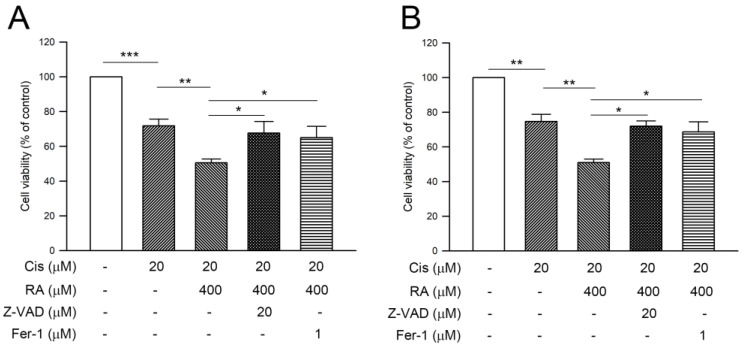
Involvement of apoptosis and ferroptosis in the cytotoxicity of cisplatin combined with RA against colon cancer cells. (**A**) DLD-1 and (**B**) LoVo cells were pretreated with Z-VAD-FMK (Z-VAD) or ferrostatin-1 (Fer-1), treated with cisplatin (Cis) or cisplatin combined with RA for 24 h, and then subjected to cell viability assessment by SRB assay. Cell viability was presented as percentage of control. *, **, and ***, *p* < 0.05, *p* < 0.01, and *p* < 0.005, respectively.

Since both apoptosis and ferroptosis were provoked in DLD-1 cells, the involvement of apoptosis and ferroptosis in the cell death triggered by the combination of RA with cisplatin was explored. As shown in Figure 6, 20 μM cisplatin alone and 20 μM cisplatin combined with 400 μM RA significantly reduced the average cell viability of DLD-1 and Lovo cells from 71.8% to 50.5% (DLD-1, Figure 6A) and from 74.7% to 51% (LoVo, Figure 6B), respectively (cisplatin alone, *p* < 0.005 vs. control; cotreatment of cisplatin and RA, *p* < 0.01 vs. cisplatin alone). The contribution of apoptosis in RA-promoted cytotoxicity was then evaluated. Pretreatment with the caspase inhibitor Z-VAD followed by combined cisplatin and RA significantly restored the average cell viability of DLD-1 cells and LoVo cells to 67.7% and 72.1%, respectively (*p* < 0.05). In parallel to apoptosis, the involvement of ferroptosis in RA-promoted cytotoxicity was assessed. Similarly, pretreatment with ferroptosis inhibitor ferrostatin-1 (fer-1) significantly elevated the average cell viability of DLD-1 cells and LoVo cells to 65.1% and 68.7%, respectively (*p* < 0.05). Collectively, these findings indicate that enhancement of apoptosis and ferroptosis is involved in the RA-promoted cisplatin-induced cytotoxicity of CRC cells.

## 4. Discussion

The present study demonstrates that RA, a polyphenolic compound found abundantly in various herbs, can potentiate the anticancer effects of cisplatin against CRC cells. We found that combining RA treatment with cisplatin synergistically inhibited cell viability and induced apoptosis in the CRC cell lines DLD-1 and LoVo. Our mechanistic investigations revealed that this enhanced cytotoxicity is mediated through the concurrent promotion of apoptosis and ferroptosis pathways.

Apoptosis, a typical form of programmed cell death, plays a crucial role in maintaining tissue homeostasis, and its dysregulation closely contributes to cancer development and progression. Consistent with previous reports on the proapoptotic effects of RA in other cancer types [15,16], we observed that RA in combination with cisplatin triggered apoptosis in colorectal cancer cells, as evidenced by the activation of caspases and the proapoptotic protein Bax, along with downregulation of the antiapoptotic protein Bcl-2. These findings align with the proposed mechanisms of RA in inducing apoptosis through the intrinsic mitochondrial pathway [17].

Notably, our study unveiled a novel mechanism by which RA potentiates the anticancer effects of cisplatin—the induction of ferroptosis. Ferroptosis is a distinct form of regulated cell death characterized by iron-dependent lipid peroxidation and the accumulation of lethal lipid peroxides [18]. Accumulating evidence indicates that ferroptosis exerts a critical tumor-suppressive function, thereby presenting novel therapeutic opportunities for targeting this pathway in cancer treatment [19]. We found that the combination treatment downregulated the expression of GPX4 and SLC7A11, key regulators of the ferroptosis pathway. GPX4 is a lipid repair enzyme that protects cells from ferroptosis by reducing lipid peroxides [20], while SLC7A11 is a component of the cystine/glutamate antiporter system that maintains intracellular levels of the antioxidant glutathione [21]. The inhibition of these ferroptosis-suppressing factors by RA and cisplatin likely contributed to the induction of ferroptotic cell death in CRC cells. Furthermore, the reversal of cytotoxicity upon treatment with ferroptosis inhibitors confirmed the involvement of this pathway. Our findings are consistent with emerging evidence indicating that the activation of ferroptosis represents an attractive therapeutic strategy for cancer treatment [22]. Moreover, several studies have demonstrated that the induction of ferroptosis can overcome drug resistance and enhance the efficacy of conventional chemotherapies [23,24]. Our findings demonstrate that RA possesses the potential to sensitize CRC cells to cisplatin-induced ferroptosis, suggesting that it may help circumvent the acquired resistance that often limits the clinical efficacy of cisplatin.

In addition to its direct cytotoxic effects, RA may also exert indirect anticancer effects by modulating the tumor microenvironment. Numerous studies have highlighted the anti-inflammatory, antioxidant, and antiangiogenic properties of RA [25,26,27], which could potentially contribute to its anticancer activity by suppressing tumor-promoting inflammation, oxidative stress, and neovascularization. However, further investigations are warranted to elucidate the potential role of these mechanisms in the context of the RA–cisplatin combination treatment for CRC.

Platinum-based anticancer drugs are commonly used for CRC treatment [28]. Cisplatin and its combinations with other chemotherapy drugs have been extensively studied for their mechanisms of action against CRC and other tumor cells. While oxaliplatin is the third platinum-based anticancer drug used clinically for the treatment of colorectal cancer, research on its predecessors has been more widespread. Our findings show that 20 μM of cisplatin alone moderately inhibits cell viability of DLD-1 and LoVo cells, and the combination of cisplatin and RA significantly enhances the cytotoxicity against these cells. These results indicate that RA enhances cisplatin’s anticancer effectiveness in vitro. However, further research using in vivo animal models is necessary to evaluate the preclinical anticancer efficacy of combining RA with cisplatin.

In conclusion, our findings reveal that rosmarinic acid can enhance the cytotoxicity of cisplatin against colorectal cancer cells through the concurrent induction of apoptosis and ferroptosis pathways. These findings highlight the potential of RA as an adjuvant therapy to potentiate the anticancer effects of cisplatin while potentially mitigating drug resistance and adverse effects. Although the in vitro cell model used in this study unveiled the mechanistic act induced by the combination of RA and cisplatin, it may not have fully recapitulated the complexity of the tumor microenvironment and the heterogeneity of tumors in patients. Future studies using in vivo xenograft models and patient-derived organoids could provide more clinically relevant insights into the therapeutic potential of this combination approach.

## Data Availability

The data that support the findings of this study are available from the corresponding author, S.-H.K., upon reasonable request.
